# Diabetes-Specific Formulae Versus Standard Formulae as Enteral Nutrition to Treat Hyperglycemia in Critically Ill Patients: Protocol for a Randomized Controlled Feasibility Trial

**DOI:** 10.2196/resprot.9374

**Published:** 2018-04-09

**Authors:** Ra'eesa Doola, Alwyn S Todd, Josephine M Forbes, Adam M Deane, Jeffrey J Presneill, David J Sturgess

**Affiliations:** ^1^ Department of Nutrition and Dietetics Mater Health Services South Brisbane Australia; ^2^ Mater Research Institute The University of Queensland Brisbane Australia; ^3^ Menzies Health Institute Griffith University Gold Coast Australia; ^4^ Glycation and Diabetes Group Translational Research Institute Brisbane Australia; ^5^ Department of Intensive Care The Royal Melbourne Hospital Melbourne Australia; ^6^ Faculty of Medicine, Dentistry and Health Sciences The University of Melbourne Melbourne Australia; ^7^ Australian and New Zealand Intensive Care Research Centre Public Health and Preventive Medicine Monash University Melbourne Australia; ^8^ Department of Anaesthesia Princess Alexandra Hospital Brisbane Australia

**Keywords:** nutrition, enteral formula, tube formula, low carbohydrate, glucose, glycemic control

## Abstract

**Background:**

During critical illness, hyperglycemia is prevalent and is associated with adverse outcomes. While treating hyperglycemia with insulin reduces morbidity and mortality, it increases glycemic variability and hypoglycemia risk, both of which have been associated with an increase in mortality. Therefore, other interventions which improve glycemic control, without these complications should be explored. Nutrition forms part of standard care, but the carbohydrate load of these formulations has the potential to exacerbate hyperglycemia. Specific diabetic-formulae with a lesser proportion of carbohydrate are available, and these formulae are postulated to limit glycemic excursions and reduce patients’ requirements for exogenous insulin.

**Objective:**

The primary outcome of this prospective, blinded, single center, randomized controlled trial is to determine whether a diabetes-specific formula reduces exogenous insulin administration. Key secondary outcomes include the feasibility of study processes as well as glycemic variability.

**Methods:**

Critically ill patients will be eligible if insulin is administered whilst receiving exclusively liquid enteral nutrition. Participants will be randomized to receive a control formula, or a diabetes-specific, low glycemic index, low in carbohydrate study formula. Additionally, a third group of patients will receive a second diabetes-specific, low glycemic index study formula, as part of a sub-study to evaluate its effect on biomarkers. This intervention group (n=12) will form part of recruitment to a nested cohort study with blood and urine samples collected at randomization and 48 hours later for the first 12 participants in each group with a secondary objective of exploring the metabolic implications of a change in nutrition formula. Data on relevant medication and infusions, nutrition provision and glucose control will be collected to a maximum of 48 hours post randomization. Baseline patient characteristics and anthropometric measures will be recorded. A 28-day phone follow-up will explore weight and appetite changes as well as blood glucose control pre and post intensive care unit (ICU) discharge.

**Results:**

Recruitment commenced in February 2015 with an estimated completion date for data collection by May 2018. Results are expected to be available late 2018.

**Conclusions:**

This feasibility study of the effect of diabetes-specific formulae on the administration of insulin in critically ill patients and will inform the design of a larger, multi-center trial.

**Trial Registration:**

Australian New Zealand Clinical Trial Registry (ANZCTR):12614000166673; https://www.anzctr.org.au/Trial/Registration/TrialReview.aspx?ACTRN=12614000166673 (Archived by WebCite at http://www.webcitation.org/6xs0phrVu)

## Introduction

### Background

Nutrition and adequate glycemic control are key management strategies associated with positive outcomes in the critically ill patient [1-4]. These components of patient management are linked since nutrition is the principal exogenous source of carbohydrate (CHO) in ventilated, tube fed patients. Nutritional formulae have the capacity to influence blood glucose control during critical illness [5]. Despite this, only a small number of studies have investigated the effect of manipulating nutritional formulae on blood glucose control in an intensive care unit (ICU) setting [6-8].

Critically ill patients experience marked changes in CHO metabolism, which contributes to hyperglycemia [9-12]. These include increased gluconeogenesis, non-insulin dependent enhanced peripheral glucose uptake, and utilization, insulin resistance and inversion of plasma glucagon to insulin ratio [11]. Hyperglycemia is prevalent in the critically ill, with up to three- quarters of patients having raised blood glucose concentrations [13]. Acute hyperglycemia is associated with various adverse outcomes including increased infectious complications, prolonged intensive care unit (ICU) and hospital stay, and an increased risk of mortality [14-16].

Historically, it was accepted as dogma that hyperglycemia was an adaptive response to stress or injury which did not require intervention [17]. This doctrine was challenged when a single center trial reported increased survival in critically ill surgical patients randomized to intensive insulin therapy aimed at maintaining blood glucose concentrations between 4.4 and 6.1mmol/L [18]. The study was pivotal to implementing strict glucose management protocols globally [19]. However, in the landmark multi-center NICE-SUGAR trial, intensive insulin therapy was linked to increased mortality compared to standard care of aiming for a blood glucose level between 6 and 10mmol/L [20]. Subsequent work has shown that it is not only hyperglycemia but also insulin-induced hypoglycemia that is strongly associated with adverse outcomes [21]. The latter has been found to be independently associated with increased mortality [22-24]. Considering that exogenous insulin affects glycemic variability [25] and, is associated with a higher risk of mortality in critically ill patients [26-28], it is appropriate to consider strategies that may facilitate reduced insulin requirements.

It has been postulated that one intervention may be substituting a more traditional exogenous nutrition with one that has a lower glycemic load and is specifically designed for hyperglycemic individuals (eg, diabetes-specific formulae [DSF]) [7]. DSF with lower CHO content has been shown to attenuate hyperglycemia and decrease glycemic variability in the non-acute setting [29-32]. However, there are limited studies evaluating the use of nutritional therapies, specifically DSF, as adjuncts to treatments for hyperglycemia in the critically ill prior to this proposed study.

Mesejo, et al. compared a high protein DSF to a standard ICU high protein formula in 50 eligible patients [7]. The aim of this open-label study was to maintain blood glucose levels between 5.5 and 11.1mmol/L through the administration of insulin as required, which is a range that varies from current practice [16,24,25]. Obese patients were excluded from participating, which has implications for the applicability of these data to countries with a greater proportion of overweight or obese patients in their ICU [33]. The investigators reported that less exogenous insulin was required to maintain acceptable glycemic control using the DSF.

De Azevedo, et al. conducted a randomized controlled trial (RCT) to assess the safety and efficacy of a CHO restrictive approach compared to intensive insulin therapy [6]. Patients allocated to the nutritional therapy group received Glucerna Select (Abbott Nutrition) and subcutaneous insulin to maintain blood glucose concentrations below 10mmol/L. The control group received an alternative formula and an insulin infusion aiming for a blood glucose target of 4.4 and 6.7mmol/L. Importantly, participants allocated to the lower target experienced a substantial increase in the frequency of hypoglycemia. These studies provide a foundation for further work investigating the role of nutrition support in the management of hyperglycemia.

In summary, glycemic control and adequate nutrition provision for critically ill patients are accepted elements of best clinical practice [34]. Hyperglycemia occurs frequently in this group, due to stress induced glucose intolerance, pre-existing diabetes mellitus and glucocorticoid therapy [18]. Management of hyperglycemia often involves exogenous insulin. Therefore, a balance of risks must be considered between hyperglycemia and treatment-induced hypoglycemia or glycemic variability [35]. The observed increase in mortality associated with insulin-induced hypoglycemia provides a strong incentive to explore managing hyperglycemia with a reduced dependency on insulin [20].

### Study Objectives

The primary aim of this study is to determine whether the administration of a DSF reduces insulin administered to critically ill patients with hyperglycemia during enteral nutrition compared to a standard liquid nutritional formula over a 48-hour period. Secondary outcomes will also be explored including the feasibility of study processes to build toward a future multi-site RCT.

A nested cohort study will also be conducted to facilitate a greater understanding of the underlying pathophysiology in critically ill patients. It will specifically seek to determine whether altered CHO and advanced glycation end product (AGE) intake, is associated with oxidative stress markers in addition to acute and chronic inflammatory biomarkers.

## Methods

### Overview

This is a prospective, double-blinded, single-center, two-phased, randomized controlled feasibility trial. It has been developed in accordance with the Standard Protocol Items: Recommendations for Interventional Trials (SPIRIT 2013) [36] and the Consolidated Standards for Reporting of Trials CONSORT Guidelines [37]. The trial complies with the Australian National Statement on Ethical Conduct in Human Research [38] and is currently being undertaken in the two adult ICUs at Mater Health in Brisbane, Australia. Both ICUs are run by the same intensive care specialists and protocols/ procedures are identical across sites but the ICUs differ in the health insurance status of their patients, which may be reflected in patient demographics and clinical case-mix [39].

This study has undergone full ethical review by the Mater Human Research Ethics Committee (Mater HREC; Approval HREC/14/MHS/55) and also has approval from The University of Queensland (UQ HREC; Approval 2014001353) due to the interventional nature of the research. It is also registered on the Australia New Zealand Clinical Trials Registry with registration number ANZCTR:12614000166673.

### Study Participants

Participants will be recruited following admission to Mater Adults Hospital or Mater Private Hospital ICU. Eligibility is determined by the following inclusion criteria – a patient is receiving exclusive enteral nutrition and requires an insulin infusion. The majority of patients with two consecutive blood glucose levels > 10mmol/L will commence on an insulin infusion as per the units’ glucose management protocol. The exclusion criteria for this study include patients under the age of 18, declined consent by the patient or legally authorized representative, or if the treating physician deems participation clinically inappropriate for any patient.

Senior ICU medical staff (consultant or advanced trainee), research assistant(s) or dietitian(s) will seek informed consent from the patient preferentially where possible, or from a legally authorized representative if required where patients lack competence. Should the latter occur, patients will be approached for their consent to be followed once alert and able. A modification to the inclusion and exclusion criteria, primarily related to lowering the threshold of insulin dose used to determine eligibility, was made after commencement of this study to optimize recruitment of appropriate patients to this feasibility study.

### Baseline Data Collection

Patient demographics including anthropometric data such as weight, height and body mass index will be collected at baseline. Additionally; admission diagnosis, acute physiology, age and chronic health evaluation (APACHE) III score, date of admission to hospital and the ICU, requirement for ventilation support, pre-existing comorbidities including pre-admission diabetic status, use of steroids and insulin at baseline will be recorded. Investigators will adopt the Patient Generated Subjective Global Assessment grading system for steroid dose which is as follows (in prednisone equivalents): low dose <10mg; moderate dose ≥10mg and <30mg and high dose ≥30mg [40].

### Randomization

Randomization between three arms will be performed initially 1:1:1 by a computer-generated sequence concealed within opaque sequentially numbered envelopes. Block randomization will be used to promote balanced patient numbers per arm. Envelopes will be prepared by a non-investigator and will include both the designated treatment arm (denoted as Feed A, Feed B or Feed C) as well as weight based recommended goal rates for administration of the formulae. Calculations of these prescribed rates are detailed in section 5. Once 12 patients have been recruited to the second intervention arm [Diason (Nutricia)], further recruitment to this treatment will cease, and the trial continues with the remaining interventional arm, Glucerna Select (Abbott Nutrition), and the control arm, Nutrison Protein Plus Multifibre (Nutricia).

### Blinding

**Figure 1:**
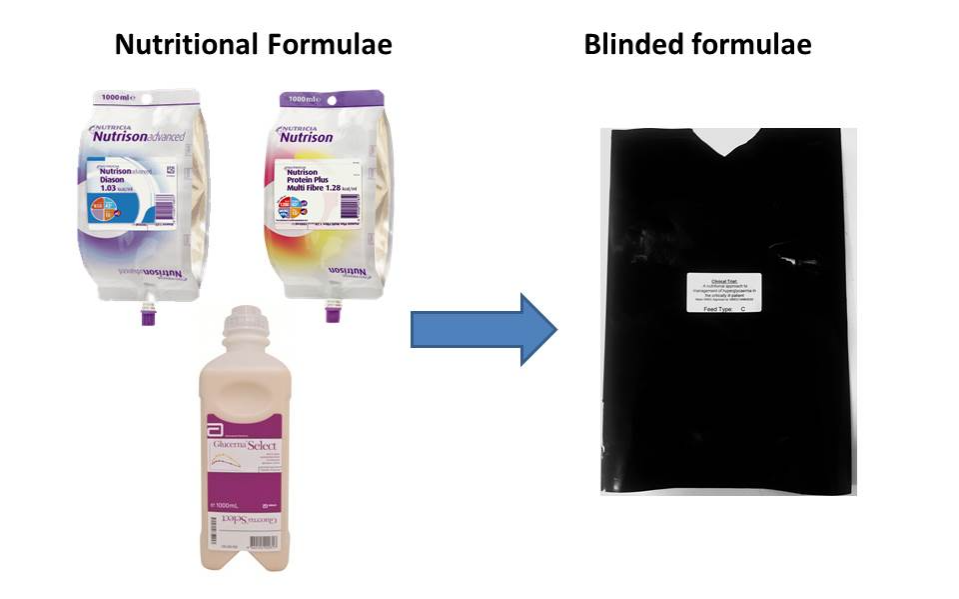
Blinding of formulae.

This study will attempt blinding of the form and appearance of the formula bags from patients and study investigators. Due to the different form factors of the formulae packaging - Glucerna Select (Abbott Nutrition) is retained in a hard-plastic bottle, while Nutricia formulae are available in soft satchels, formula containers will be placed within an opaque masking bag with an opening in the bottom to allow for connection to giving sets (Figure 1). Blinding of the formulae will be carried out by a non-investigator and the concealed bags marked with the study details and treatment arm. These will be placed into corresponding storage containers for nursing staff to access.

### Study Design

Following consent participants will be randomized to either control, which is the unit’s standard nutritional formula, Nutrison Protein Plus Multifibre (Nutricia), or the intervention, which is a low CHO, low glycemic index DSF, Glucerna Select (Abbott Nutrition). For the purpose of the nested cohort study, a second intervention arm using a different low glycemic index DSF will be used [Diason (Nutricia)]. Refer to Table 1 for a complete nutritional breakdown of these products. Varying advanced glycation end-product content of the differing DSF necessitates the third arm as part of this study. Randomization to the second intervention arm will be ceased once a complete set of blood and urine samples are available for a minimum of 10 participants in each group, with 12 patients set as the target recruitment to account for retracted/declined consent, incomplete sample sets in the instances of extubation or discharge to the ward prior to retrieval. These samples will be collected from all patients at randomization and 48 hours later. Once this has been achieved, randomization will revert to control [Nutrison Protein Plus Multifibre (Nutricia)] and intervention [Glucerna Select (Abbott Nutrition)] groups only. Collection of urine and blood samples for biomarker assay will also be discontinued at this point.

Each envelope will include detailed instructions for clinical staff regarding nutritional formula type labeled as Feed A, B or C and a protocolized delivery rate. The rate has been determined to minimize difference in protein provision in an effort to mitigate confounding factors based on studies identifying the protein as having a protective effect [2]. Once group allocation has occurred, participants will remain on their specified study formula as long as tube feeding continues in ICU or at the discretion of the treating clinician. If the participant were to withdraw from the study, clinical care would revert to usual practice such that the formula will be changed over to what would have otherwise been used had the trial not taken place (Nutrison Protein Plus® or Diason® - Nutricia). Due to the pragmatic nature of this study, all other aspects of care will occur as per routine clinical practice. That is, rates of feeding and treatment of intolerance to enteral nutrition will be as per the unit’s standardized enteral feeding protocol.

### Outcome Measures

#### Primary Outcome Measures

The primary outcome of this study is the difference in total amount of insulin per 24 hours administered to patients following commencement of the study formula until the 48-hour time period [7]. Pre-study insulin use will also be recorded from the patient charts for the 12 hours preceding randomization.

#### Secondary Outcome Measures

A range of secondary measures will be explored as outlined below.

**Table 1:** Nutritional composition of study feeds. RE: retinol equivalent. α-TE: alpha tocopherol equivalent. NE: niacin equivalent.

Formula Composition		Nutrison Protein Plus Multifibre (per 1000mL/per kcal/1000mL)	Glucerna Select (per 1000mL)	Diason (per 1000mL)
Energy (kJ)		5350/4180	4170	4350
Protein (g)		63/49 (20%E^a^)	50 (20%E^a^)	43 (17%E^a^)
Carbohydrate (g)		141/110 (44%E^a^)	75 (28.6%E^a^)	113 (44%E^a^)
Fat (g)		49/38 (34%E^a^)	54 (48%E^a^)	42 (36%E^a^)
Saturated Fat(g)		13/10.2	4	5
Monounsaturated Fat(g)		27/21	Not specified	30
Polyunsaturated Fat(g)		9/7	Not specified	7
Fiber (g)		15/12	21	15
**Micronutrients (Minerals and Vitamins)**				
	Sodium (mg)	1110/867	940	1000
	Potassium (mg)	1680/1313	1300	1500
	Calcium (mg)	900/703	700	800
	Phosphorus (mg)	900/703	650	720
	Magnesium (mg)	280/219	210	230
	Chloride (mg)	800/625	1250	1250
	Vitamin A (mcg RE)	1020/797	580	820
	Vitamin D (mcg)	17/13	9.3	7
	Vitamin E (mg α-TE)	16/13	19	25
	Vitamin K (mcg)	66/52	100	53
	Vitamin C (mg)	130/102	110	150
	Thiamine (mg)	1.9/1.5	1.5	1.5
	Riboflavin (mg)	2/1.6	1.8	1.6
	Niacin (mg NE)	23/18	17	18
	Vitamin B6 (mg)	2.1/1.6	2.1	1.7
	Vitamin B12 (mcg)	2.6/2.0	3	5
	Folic Acid (mcg)	330/258	250	380
	Pantothenic Acid (mg)	6.6/5.2	7.5	5.3
	Biotin (mcg)	50/39	40	40
**Trace Elements**				
	Iron (mg)	20/15.5	13	16
	Zinc (mg)	15/11.7	12	12
	Manganese (mg)	4.1/3.2	3.5	3.3
	Copper (mcg)	2250/1758	1400	1800
	Iodine (mcg)	170/133	110	130
	Molybdenum (mcg)	130/102	100	100
	Selenium (mcg)	71/56	50	75
	Chromium (mcg)	83/65	85	120
Osmolality (mOsmol/kg H_2_ O)		360	450	360

^a^Percentage of total calories; E: energy.

##### Scientific Measures

Many of the quantitative data measures will be collected within the first 48 hours post randomization including glycemic variability [41], tolerance of enteral nutrition and ability to meet nutritional requirements if there were no clinical contraindications [42]. Glycemic variability will be measured using routinely collected glucometer blood glucose readings which are taken between hourly and 4 hourly, depending on previous blood glucose and insulin rate. The mean and standard deviation of these values will be utilized to calculate the coefficient of variation which forms a surrogate marker of variability [43]. Tolerance of prescribed nutrition will be measured through establishing the incidence of diarrhea commencing post-randomization, the collection of gastric residual volumes [44,45] and potential subsequent use of prokinetics as well as ability to meet recommended goal rates of nutrition in the absence of any clinical contraindication. Goal rates are calculated based on protein requirements of 1.2-1.5g/kg body weight (BW) or adjusted ideal body weight (AIBW) and 25-30kcal/kg BW/AIBW [46]. Adjusted ideal body weight is to be used in the instance a patient has a body mass index greater than 25kg/m^2^.

##### Process Measures Used to Assess Feasibility

Suitable patients, based on the inclusion and exclusion criteria, will be identified by clinical staff and subsequently eligibility and consent verified by the trial research staff. The eligibility criteria used in this study methodology will be assessed for sufficiency to ensure patients are appropriately screened, consented and recruited. Furthermore, data obtained from this study will be used to determine whether a pre-defined period of time expected on nutrition support is required as a component of the inclusion criteria. This will be assessed by the proportion of patients reaching the 48-hour mark on nutrition support. Recruitment rates will be determined by the number of eligible patients who consent in their own right or consent has been obtained from their legally authorized representative. Refusal of consent at either of these points will be recorded with reasons if provided. Based on predictive modelling of patient numbers for Mater Health ICUs to date it is expected that patients will be recruited over a two-and-a-half-year period. This can be extended for a further 6 months dependent upon recruitment rates. The pilot study intends to recruit a minimum of 54 patients.

#### Tertiary Outcome Measures

Length of ICU and hospital stay data as well as mortality outcomes will be investigated at 28 days through a follow up phone call in the instance a patient has been discharged from the hospital, or by access to medical records in the event of death before hospital discharge. Within scripted questions, data related to weight, weight history, diet and functional capacity pre and post ICU admission, will be collected, based on adaption from a validated malnutrition assessment tool (Patient-Generated Subjective Global Assessment) [47]. Blood glucose control including insulin/oral hypoglycemic use pre and post ICU admission will also be explored. The planned sub-study aims to analyze blood and urine samples to establish differences in acute and chronic inflammatory markers, cortisol, oxidative stress and anti-oxidant capacity between baseline and 48 hours later while on differing nutritional products [48-53]. Blood samples will be retrieved through existing arterial lines, centrifuged within 45 minutes and stored at negative eighty degrees Celsius until batch analysis. Urine samples will be taken and aliquots stored under the same conditions.

### Statistical Considerations and Data Analysis

#### Sample Size

**Figure 2:**
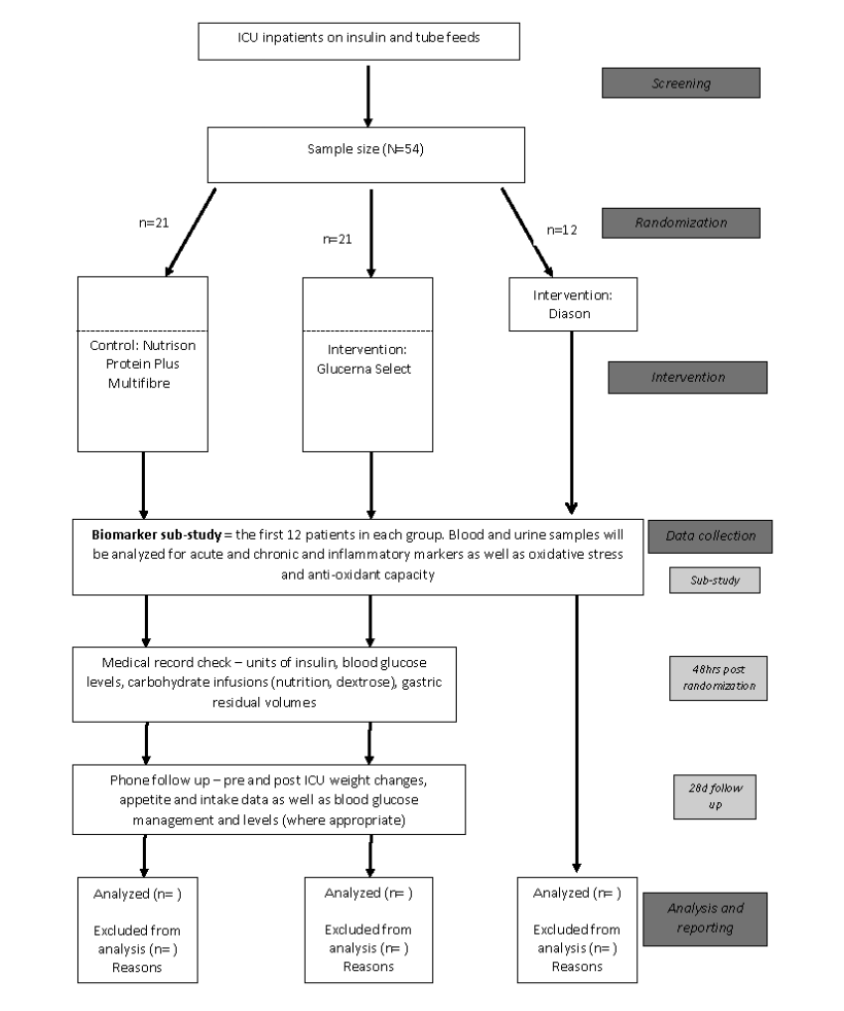
Study flow diagram of recruitment, randomization and study conduct. Once consented patients will be randomized on a 1:1:1 ratio until there are 12 patients who have complete blood and urine sample sets from each group to form the biomarker sub-study. Thereafter the study will proceed with two arms as indicated. Finalized patient numbers have not been provided as this is a feasibility study.

There is a minimum target recruitment of 54 patients for this pilot feasibility trial (refer to Figure 2). Two arms of this study will require nineteen patients each to detect a statistically significant difference (alpha 0.05) with a power of 80% based on a median difference of 21.5 units of insulin per day and a common standard deviation of 22.5 units. This has been modeled after the enteral formula study by Mesejo, et al. (2003). For the third arm, a sample size of 10 patients will be randomized for the nested cohort study investigating relevant biomarkers. A 10% buffer of two patients per arm, in the instance of retracted consent or earlier than expected cessation of nutrition, has been accounted for in the target sample size of 54 patients with 21 patients in the primary study (two arms) and 12 in the third arm forming part of the nested cohort study i.e. 2 additional patients per arm.

#### Data Analysis Plan

The analysis will be performed using R commander software or equivalent. Descriptive statistics (frequency, means and standard deviations, medians, interquartile ranges and full ranges) will be calculated for relevant demographic and baseline variables as well as for trial endpoints. Linear, logistic and time-to-event analyses will be performed using relevant data, returning point estimates of effect with associated 95% confidence intervals. Cross sectional analyses in cohort study data with equal follow-up time per subject will be analyzed with linear (for continuous outcomes) or logistic models (for categorical outcomes). Univariable time to event analyses will use Kaplan-Meier curves and differences will be tested using log rank tests.

Adjusted time-to-event analyses will use Cox proportional hazards models, with the assumed proportionality of the hazard subject to verification. Analyses using data collected over time within individual patients will be modeled using methods that account for patient-level clustering (generalized estimating equation methods or mixed linear or logistic models). In the event of non-negligible proportions of missing data, sensitivity analyses will present both the results of complete case analyses and multiple imputation analyses. Linear regression analyses will be used to analyze results from the nested cohort study to determine the contribution of AGE intake (total feed delivered accounting for formula content), AGE output (urine output accounting for AGE level in the urine) and insulin dose (area under the curve) on sRAGE levels.

## Results

Recruitment to this study commenced in February 2015 and has an estimated completion date for data collection by May 2018. Results are expected to be available in late 2018.

## Discussion

### Expected Outcomes and Potential Significance

The findings of this feasibility study will be used to refine the design of a larger multi-center trial identifying protocol strengths and limitations and importantly returning preliminary study effect estimates for sample size estimation. A more detailed budget will also be established using data from this initial study.

The pragmatic approach of negligible alterations to the pre-existing ICU policies and procedures regarding glucose management is intentional, aiming for this study to proceed with minimal disruption to standard ICU clinical work routines. If successful, a subsequent larger multi-center trial derived from the present proposed pilot study may clarify the efficacy of an intervention altering the composition of enteral nutrition in ICU patients as a standalone therapy for dysregulated glucose management in ICU patients.

### Conclusion

Treatment of hyperglycemia, predominantly involving insulin therapy, remains controversial in the critically ill. While there is evidence to indicate diabetes specific enteral nutrition formulae are beneficial in reducing blood glucose levels in general patients with diabetes, at both a ward and community level, limited evidence supports this practice in the intensive care setting. At present, modification of the composition of enteral nutrition is rarely considered to be an efficacious mode of therapy for glucose control despite its significant contribution to total carbohydrate intake. This pilot study intends to investigate the feasibility and efficacy of diabetic specific nutrition formulations as an additional therapeutic measure in glucose management in the ICU.

## Abbreviations

AIBW: adjusted ideal body weight
AGE: advanced glycation end product
APACHE: acute physiology, age and chronic health evaluation
BW: body weight
CHO: carbohydrate
DSF: diabetes-specific formula
HREC: Human Research Ethics Committee
ICU: intensive care unit
RCT: randomized controlled trial
